# Effect of Cocoa Polyphenolic Extract on Macrophage Polarization from Proinflammatory M1 to Anti-Inflammatory M2 State

**DOI:** 10.1155/2017/6293740

**Published:** 2017-06-28

**Authors:** Laura Dugo, Maria Giovanna Belluomo, Chiara Fanali, Marina Russo, Francesco Cacciola, Mauro Maccarrone, Anna Maria Sardanelli

**Affiliations:** ^1^Department of Medicine, Campus Bio-Medico University of Rome, Via A. del Portillo 21, 00128 Roma, Italy; ^2^Department of Biomedical, Dental Sciences and Morphological and Functional Images, University of Messina, Via Consolare Valeria, 98125 Messina, Italy; ^3^European Center for Brain Research, Santa Lucia Foundation IRCCS, Rome, Italy; ^4^Department of Basic Medical Sciences, Neurosciences and Sense Organs, University of Bari “Aldo Moro”, P.zza G. Cesare, 11, 70124 Bari, Italy

## Abstract

Polyphenols-rich cocoa has many beneficial effects on human health, such as anti-inflammatory effects. Macrophages function as control switches of the immune system, maintaining the balance between pro- and anti-inflammatory activities. We investigated the hypothesis that cocoa polyphenol extract may affect macrophage proinflammatory phenotype M1 by favoring an alternative M2 anti-inflammatory state on macrophages deriving from THP-1 cells. Chemical composition, total phenolic content, and antioxidant capacity of cocoa polyphenols extracted from roasted cocoa beans were determined. THP-1 cells were activated with both lipopolysaccharides and interferon-*γ* for M1 or with IL-4 for M2 switch, and specific cytokines were quantified. Cellular metabolism, through mitochondrial oxygen consumption, and ATP levels were evaluated. Here, we will show that cocoa polyphenolic extract attenuated in vitro inflammation decreasing M1 macrophage response as demonstrated by a significantly lowered secretion of proinflammatory cytokines. Moreover, treatment of M1 macrophages with cocoa polyphenols influences macrophage metabolism by promoting oxidative pathways, thus leading to a significant increase in O_2_ consumption by mitochondrial complexes as well as a higher production of ATP through oxidative phosphorylation. In conclusion, cocoa polyphenolic extract suppresses inflammation mediated by M1 phenotype and influences macrophage metabolism by promoting oxidative pathways and M2 polarization of active macrophages.

## 1. Introduction

Monocyte-derived macrophages play a crucial role in inflammation, host defense, and tissue repair [1, 2]. Macrophages have important pathogenic roles in many chronic diseases, such as asthma, inflammatory bowel disease, atherosclerosis, rheumatoid arthritis, and fibrosis [2–4]. Many studies have attempted to simulate the process of monocyte to macrophage differentiation in vitro, through the culture of monocytes with or without addition of different cytokines [5]. Phenotypic and functional flexibility is a key property of macrophages [6–9]. In vivo, human macrophages can be classified into two categories according to their activation states. A classical M1 phenotype and an alternative M2 phenotype have been characterized on the basis of their reactions to different stimuli. Over the last few years, considerable progress has been made toward characterization of epigenetic mechanisms, transcription, and posttranscriptional factors regulating macrophage polarization [10]. M1 activation is drived by the cytokine interferon- (IFN-) *γ* and the activation of Toll-like receptors (TLRs) by lipopolysaccharide (LPS), while M2 activation is triggered by interleukin- (IL-) 4 [6]. It is now known that other mediators, besides IL-4, as well as IL-13 can drive M2 polarization [11, 12]. However, M1 and M2 phenotypes are two limits of a variety of functional states, which make up the complexity of macrophage flexibility [13–15]. M1 macrophages are characterized by high production of proinflammatory cytokines (including tumor necrosis factor- (TNF-) *α*, IL-1*β*, IL-6, and IL-12) and reactive nitrogen and oxygen species (RNS, ROS), promoting T helper- (h-) 1 cells that express IFN-*γ* response and having strong tumoricide and microbicide activity [15]. On the other hand, M2 macrophages have immune regulatory function, an efficient phagocytic activity and are characterized by high levels of scavenging molecules, by the production of ornithine and polyamines through the arginase pathway and by the expression of mannose and galactose receptors [9].

The onset of a pathological state is frequently associated with dynamic changes in macrophage stimulation; in fact, M1 macrophages are involved in initiating and sustaining inflammation while M2 macrophages are linked with resolution or chronic inflammation [7, 16]. Polarized phenotypes are reversible in vitro and in vivo [17–19]. Modulation of macrophage function is an off-target effect for various therapeutic agents such as STAT3/STAT6 inhibitors, imidazoquinolines, peroxisome proliferator-activated receptor- (PPAR-) *γ* agonists, and CD40 [6].

Bioactive food compounds, such as the polyphenols, have recently gained consideration for their anti-inflammatory properties, which might have an impact on macrophage phenotype favoring an alternative M2 anti-inflammatory state. It was demonstrated that pomegranate polyphenols dose dependently attenuated macrophage response to M1 proinflammatory activation in the J774.A1 macrophage-like cell line [20]. This was supported by a significant decrease in the proinflammatory cytokine secretion in response to stimulation by IFN-*γ* and LPS and by a significant increase in IL-10 secretion promoting the differentiation of a M2 anti-inflammatory phenotype [20].

Various in vitro studies have attributed downregulation of the inflammatory response to cocoa polyphenols. Cocoa, a product derived from the beans of *Theobroma cacao* plant, is a rich source of monomeric polyphenolic antioxidants, mainly epicatechin and catechin, and various polymers derived from these monomers, identified as procyanidins [21]. Cocoa has a potent antioxidant capacity, as compared with other products [22], related to flavonoid content [23]. In addition to their potent antioxidant characteristic, cocoa polyphenols were shown to possess remarkable anti-inflammatory properties [24]. In vitro, flavonoids present in cocoa decreased the production of inflammatory cytokines (TNF-*α*, IL-6, and IL-1*β*), ROS, and RNS, in LPS-stimulated macrophages [25]. In a healthy population of Southern Italy, regular intake of dark chocolate was inversely associated to serum C-reactive protein level [26, 27]. Furthermore, cocoa treatment was shown to be effective in the prevention of cardiovascular diseases and in the modulation of blood pressure in animal and human studies [20–30]. With regard to its effect in inflammatory conditions, they still need to be further explored [31]. In animal models of diseases, such as inflammatory bowel disease, arthritis, and colitis, cocoa polyphenols reduced both the inflammatory response and the oxidative stress [32, 33]. In this context, cocoa has been shown to modulate the immune system, in particular, the systemic and intestinal immune responses and the inflammatory innate response [34].

About the mechanism by which cocoa flavonoids modulate immune function, it has been suggested that they reduce redox sensitive nuclear factor kappa-light-chain-enhancer of activated B cell (NF-kB) activation and, consequently, the expression of many genes involved in cytokine secretion [24, 35, 36]. They can also directly interact with gene expression factors and cell signaling involved in cytokine secretion, such as activator protein 1 (AP-1) [37] and signal transducer and activator of transcription 4 (STAT4) [38]. Further studies are needed to elucidate the interactions between cocoa and the redox-sensitive signalling pathways involved in the expression of many genes and, consequently, in several cell functions, such as the immune response.

The identification of mechanisms and molecules associated with macrophage flexibility and polarization will provide a basis for macrophage-centered diagnostic and therapeutic strategies. Although previous studies demonstrated that cocoa polyphenols possess anti-inflammatory effects, to the best of our knowledge, a research focused on the direct effect of the cocoa polyphenols on macrophage inflammatory phenotype has never been performed.

In this study, we have investigated the hypothesis that a cocoa polyphenolic extract may influence the macrophage polarization through the metabolic switch promoting a M2 anti-inflammatory state.

## 2. Materials and Methods

### 2.1. Cocoa Polyphenol Extraction

Cocoa beans, commercially available locally, was originated from Ghana, West Africa. Polyphenol extraction was carried out as previously described [26] with some modifications as shown below. To remove lipids, cocoa beans (3 g) were ground to a powder with quartz sand, then was vortexed in 10 ml of hexane, and centrifuged for 5 minutes at 800 ×g and 4°C. Hexane extraction was repeated for three times. Subsequently, nonfat cocoa grain was dissolved in 10 ml of methanol/water solution, 70 : 30 (*v/v*), and polyphenols were extracted by three centrifugations at 800 ×g and 4°C for 5 minutes. Cocoa polyphenolic extract was filtered and evaporated until dry in a rotary evaporator. Finally, samples were dissolved in methanol/water/acetic acid solution, 70 : 28 : 2 (*v/v/v*).

### 2.2. Determination of Phenolic Compounds and Total Antioxidant Capacity in Cocoa Polyphenolic Extract

Phenolic content of cocoa extract was measured by a modified Folin-Ciocalteu method using gallic acid as standard [39]. The diluted 1 : 40 (*v/v*) aqueous solution of extract (20 *μ*l) was mixed with Folin-Ciocalteu reagent (100 *μ*l) and was incubated with Na_2_CO_3_ 1.89 M for 2 hours. The absorbance was measured at 765 nm by a multifunctional microplate reader (Infinite^M^ 200 Pro, TECAN, Italy) in the samples dispensed in triplicate in a 96-well cell culture plate (Greiner Bio-One, Germany). The results were expressed as mg of gallic acid equivalent (GAE)/l.

Total antioxidant capacity of cocoa extract was measured by using the Trolox equivalent antioxidant capacity (TEAC) assay [40]. Briefly, 10 *μ*l of cocoa polyphenolic extract was mixed with 200 *μ*l of ABTS^+^ solution in a 96-well cell culture plate and the absorbance was recorded at 734 nm for 90 seconds by a multifunctional microplate reader (Infinite^M^ 200 Pro, TECAN, Italy). TEAC values were calculated from the Trolox standard curve (60–300 *μ*M).

### 2.3. RP-HPLC/PDA/MS Analysis of Cocoa Polyphenolic Extract

Cocoa polyphenolic extracts, before the RP-HPLC/PDA/MS analysis, were filtered to a 0.45 *μ*m Acrodisc filter (Pall Life Sciences, Ann Arbor, MI, USA).

HPLC analyses, performed on 2 *μ*l of the injection volume, were carried out as previously described [40]. In particular, the mobile phase contained water (Sigma-Aldrich) /formic acid (Riedel-de Haën, Hanover, Germany) (99.9 : 0.1, (*v/v*)) (solvent A) and acetonitrile (Sigma-Aldrich)/formic acid (99.9 : 0.1, (*v/v*)) (solvent B), and the stepwise gradient profile was as follows: 0 minutes, 5% B; 30 minutes, 30% B; 35 minutes, 100% B; and 36 minutes, 0% B. Flow rate was 0.7 ml/minute. An SPD-M20A UV detector and an LCMS-2020 were employed. Data were obtained by a photodiode array (PDA) detector in the range 190–400 nm. Time constant was 0.64 s and sample frequency 1.5625 Hz. MS acquisition was carried out using ESI interface (Shimadzu), in negative mode by the LCMS solution Ver. 3.30 software (Shimadzu). 200 *μ*l of the whole LC flow rate was directed to the interface, and the total flow was switched to waste (500 *μ*l) and to interface (200 *μ*l) by means of a flow splitter. ESI conditions: mass spectral range, *m/z* 100–1200; even time, 1 s; scan speed, 1154 amu/s; nebulizing gas (N_2_) flow, 1.5 l/minute; ESI temperature, 300°C; heat block, 300°C; DL (desolvation line) temperature, 250°C; DL voltage, 34 V; probe voltage, +4.5 kV; Qarray voltage, 1.0 V; and detection gain, 1.05 kV.

### 2.4. Cell Culture and Differentiation

The THP1 cell line was acquired from ATCC and cultivated in RPMI 1640 medium (Lonza, Belgium) supplemented with 10% fetal bovine serum (FBS; Lonza, Belgium), 1 mM L-glutamine (Sigma-Aldrich, Milan, Italy), 100 U/ml penicillin, and 100 *μ*g/ml streptomycin (Sigma-Aldrich, Milan, Italy) in 5% CO_2_ at 37°C to give a final concentration of approximately 2 × 10^5^cells/ml. THP-1 cells were differentiated for 72 hours into macrophages (M0) by stimulation with 100 ng/ml phorbol 12-myristate 13-acetate (PMA, Sigma-Aldrich, Milan, Italy). After macrophage differentiation, cells were cultured for another 24 hours with either LPS (1 *μ*g/ml) + INF-*γ* 20 ng/ml to generate M1-macrophages, or with IL-4 (20 ng/ml) to generate M2-macrophages [41], in the absence or in the presence of cocoa polyphenolic extract at different concentration expressed as *μ*M GAE as indicated in the figures.

### 2.5. Cell Viability

M1-polarized cells were seeded in a multiwall plate at concentration of 80000 cells/well and incubated for 24 hours in the absence (control) and in the presence of cocoa polyphenolic extract (0.1–100 *μ*Μ GAE). Successively, cell culture medium was discarded, and each well was washed with 200 *μ*l Hank's balanced salt solution (HBSS+, Gibco, Waltham, MA, USA). MTT solution (0.5 mg/ml, 100 *μ*l Sigma-Aldrich, Milan, Italy) was added to cells in each well, and the plate was incubated at 37°C + 5% CO_2_ for about 3 hours, until MTT formazan crystals were visible in the culture liquid. Then MTT solution was removed, and dimethyl sulfoxide (DMSO, Sigma-Aldrich, Milan, Italy) (70 *μ*l/well) was added to each well for dissolving the formazan crystals. Optical density (OD) was measured at 565 nm using a multifunctional microplate reader (Infinite^M^ 200 Pro, TECAN, Italy). Viability was calculated as the ratio of the mean of OD obtained for each condition to that of control (absence of cocoa polyphenolic extract) condition.

### 2.6. Cytokine Secretion

The levels of cell-released TNF-*α*, IL-6, IL-1*β*, IL-12, and IL-10 were measured in the harvested supernatants by using an ELISA Ready-Set-Go kit (eBioscience, San Diego, CA, USA), following the manufacturer's instructions. Optical density was determined using the microplate reader Infinite^M^ 200 Pro, TECAN (Italy).

### 2.7. Evaluation of Mitochondrial Respiratory Activity

Mitochondrial oxygen consumption was measured polarographically with a Clark-type oxygen electrode in a thermostated gas-tight chamber (Hansatech Instruments, Norfolk, UK) [42, 43]. Measurements of substrate-supported respiration were carried out in intact exponentially growing cells. The culture medium was changed 1 day before the assays. Cells were trypsinized, centrifuged, and resuspended at 2 × 10^6^ cells/ml in 0.137 M NaCl, 5 mM KCl, 0.7 mM Na_2_HPO_4_, and 25 mM Tris–HCl, pH 7.4. An aliquot of cell suspension was used for counting and protein determination. The cells were then transferred into the polarographic chamber. For the measurement of respiration rates by exogenous substrates, after full uncoupling of the endogenous respiration of intact cells with 30 *μ*M dinitrophenol (DNP), digitonin (30 *μ*g/10^6^ cells) was added. After 2 minutes, added respiratory substrates were as follows: pyruvate (5 mM)/malate (2.5 mM) for complex I, succinate (5 mM) for complex II + complex III, in the presence of inhibitor of complex I, and rotenone (200 nM) and inhibitor of complex III antimycin A (13 nM). All amounts of oxygen depletion were normalized to cellular protein content.

### 2.8. ATP Measurement

Cellular ATP levels under basal conditions were measured by a luminometer (Infinite^M^ 200 Pro, TECAN, Italy) with the ATP lite kit (Perkin Elmer, Waltham, MA) through a luciferin-luciferase reaction system, according to the manufacturer's instructions. Macrophage differentiated from THP-1 cells M0, and M1/M2 polarized cells were collected from Petri dishes with 0.05% trypsin, 0.02% EDTA, pelleted by centrifugation at 500 ×g, and washed in phosphate-buffered saline (PBS), pH 7.4. For each assay, 60000 cells in multiwell plate were used. For the evaluation of ATP content under strict glycolytic conditions, M0, M1, and M2 were incubated for 5 hours at 37°C in both in the presence of rotenone (1 mmol/l) that of antimycin A (1 mmol/l), [44]. An ATP calibration curve was made using known concentration ATP solutions. An aliquot of cell lysate was employed for protein content quantification.

### 2.9. Statistical Analysis

In order to evaluate the dose-response effects, we applied the one-way repeated measures analysis of variance, whereas the differences between treated and untreated M0, M1, and M2 cells were analyzed by the two-way repeated measures ANOVA (two-factor repetition). Student-Newman-Keuls Method was used as all pairwise multiple comparison procedure. The results are given as mean ± SD. Values of *p* ≤ 0.05 were chosen as the criteria for statistically significant difference.

## 3. Results

### 3.1. Cocoa Polyphenol Extract Characterization: Total Phenolic Content, Antioxidant Capacity, and Chemical Composition

The total phenolic content and the antioxidant capacity of cocoa polyphenolic extract were determined by Folin-Ciocalteu and TEAC assays, respectively. The results presented in Table 1 suggest that the values of total phenolic content, expressed as mg gallic acid equivalent (GAE)/l, reflect the antioxidant activity, expressed as mM Trolox equivalent (TE)/g fresh mass, of cocoa extract.

**Figure 1:**
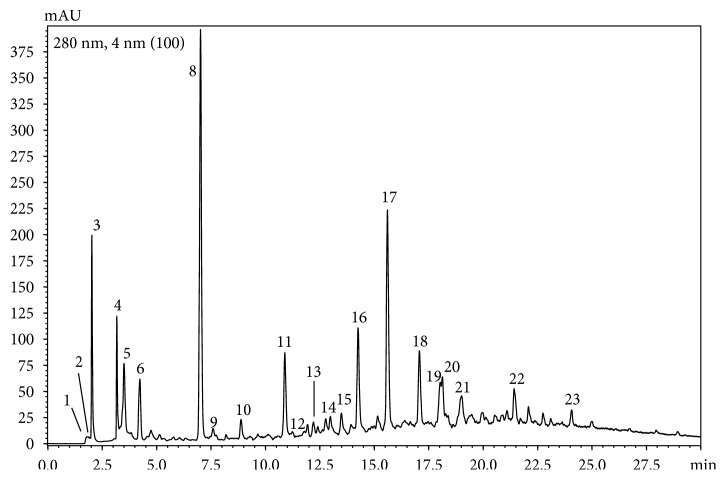
RP-HPLC-PDA chromatogram (extracted at 280 nm) of cocoa polyphenol extract. The chromatogram shows the phenolic compounds present in the cocoa extract. For peak identification, see Table 2. Almost 18 compounds were identified by LC-MS as described under the Materials and Methods.

Through HPLC-PDA-MS qualitative analysis, chemical composition of cocoa polyphenol extract was determined (Figure 1, Table 2). As can be seen from the chromatogram reported in Figure 1, the sample analyzed contains 23 bioactive molecules. Phenolic acids, flavan-3-ols, alcaloids, and cyanidins represent the class of bioactive molecules present in the sample of our interest.

### 3.2. Cocoa Polyphenols Increased Viability and Attenuate Macrophage Inflammatory Response

To test the effect of cocoa polyphenols on cell viability and inflammatory response, THP-1 macrophages were incubated with increasing concentration of CPE, expressed as *μ*Μ GAE (0.1–100 *μ*Μ GAE), earlier to exposure to M1 activation induced by the INF-*γ* and LPS.

**Figure 2:**
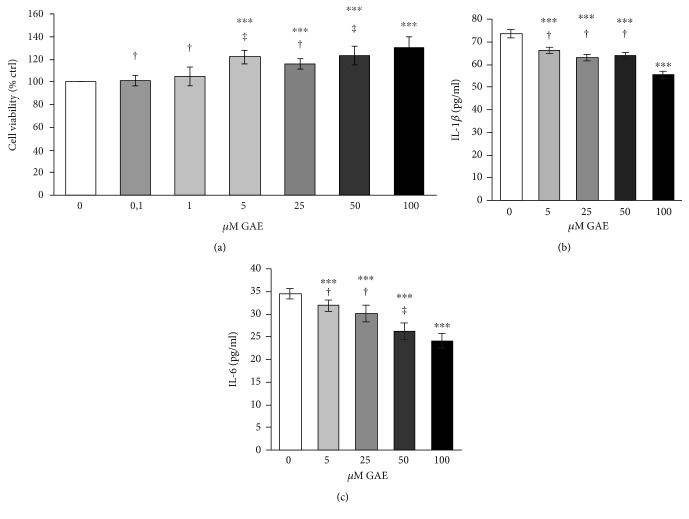
Effect of cocoa polyphenolic extract on cell viability and on macrophage M1 inflammatory state. THP-1 macrophages were stimulated with 20 ng/ml INF-*γ* **+** 100 ng/ml LPS as described under the Materials and Methods and incubated in the presence of cocoa polyphenolic extract expressed (CPE) in *μ*Μ GAE as indicated. (a) Cell viability assayed by MTT methods was expressed as % of control (0 *μ*Μ GAE). (b) Levels of IL-1*β* and (c) IL-6, determined by ELISA assay, were expressed as pg standard/ml. White bars indicate the controls, and grey scale the increasing concentrations of GAE. All data represent the means of 3/5 replicates ± standard deviation. One-way repeated measures analysis of variance, followed by Student-Newman-Keuls method. ^∗∗∗^*p* < 0.001 cells treated with GAE versus untreated cells; ‡*p* < 0.01 and †*p* < 0.001 other concentrations versus 100 *μ*Μ GAE.

As presented in Figure 2(a), cocoa extract at up to 100 *μ*Μ GAE concentration failed to display toxicity toward differentiated cells. Indeed, after exposure to higher concentrations of cocoa extract from 5 *μ*Μ up to 100 *μ*Μ GAE for 24 hours, cell viability was significantly increased. In particular, cocoa extract at 100 *μ*Μ GAE increased cell viability of about 30% compared to control (Figure 2(a)).

Later, for investigating the effect of cocoa polyphenolic extract on inflammatory response, production of proinflammatory cytokines, IL-1*β* (Figure 2(b)) and IL-6 (Figure 2(c)), was quantified in M1 inflammatory macrophages in a dose-dependent manner. A concentration range from 5 *μ*Μ up to 100 *μ*Μ GAE was selected because it showed a significant increase in cell viability (Figure 2(a)). The results showed in Figures 2(b) and 2(c) suggest that cocoa extract attenuated macrophage response to M1 proinflammatory activation through the reduction of about 30% (at 100 *μ*Μ GAE) of proinflammatory cytokines.

### 3.3. Cocoa Extract Influences a Macrophage M1 to M2 Phenotypic Switch

**Figure 3:**
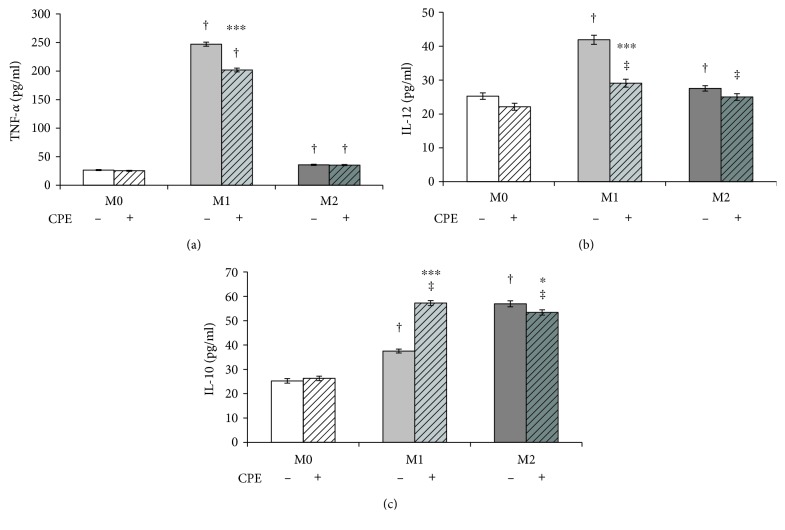
Effect of cocoa polyphenolic extract on M1/M2 phenotype switch. Macrophage-differentiated THP-1 (M0) cells and polarized (M1, M2) cells as described under the Materials and Methods were incubated in the absence (−) and in the presence (+) of cocoa polyphenolic extract (CPE) which concentration was expressed as 100 *μ*M GAE. Levels of (a) TNF-*α*, (b) IL-12, and (c) IL-10 expressed as pg/ml were measured in the collected incubation medium. Error bars represent data from 3 independent experiments: two-way repeated measures ANOVA (two-factor repetition), followed by Student-Newman-Keuls method. ^∗^*p* < 0.05, ^∗∗∗^*p* < 0.001, cells treated with CPE versus untreated. Comparisons M1 versus M2 within treatment: ‡*p* < 0.01 and †*p* < 0.001.

To investigate the effect of cocoa polyphenolic extract (CPE) on macrophage alternative M2 phenotype, the secretion of the proinflammatory and anti-inflammatory cytokines was then determined and compared to the control basal level in untreated cells. CPE significantly decreased macrophage response to M1 activation through the reduction of the secretion of proinflammatory cytokines TNF-*α* (Figure 3(a)) and IL-12 (Figure 3(b)) in M1 phenotype by up to 20% and 30%, respectively, compared to untreated cells. Furthermore, CPE increased by up 47% the release of the anti-inflammatory IL-10 (Figure 3(c)) in M1 macrophages. Cocoa extract did not exert any effect on cytokine secretion in M0 and M2 macrophages. Taken together, these results indicate that cocoa extract not only reduces inflammation in M1 cells but also promotes macrophage polarization toward the M2 alternative phenotype.

### 3.4. Effect of Cocoa Polyphenol Extract on M1/M2 Phenotype through Metabolic Switch

**Figure 4:**
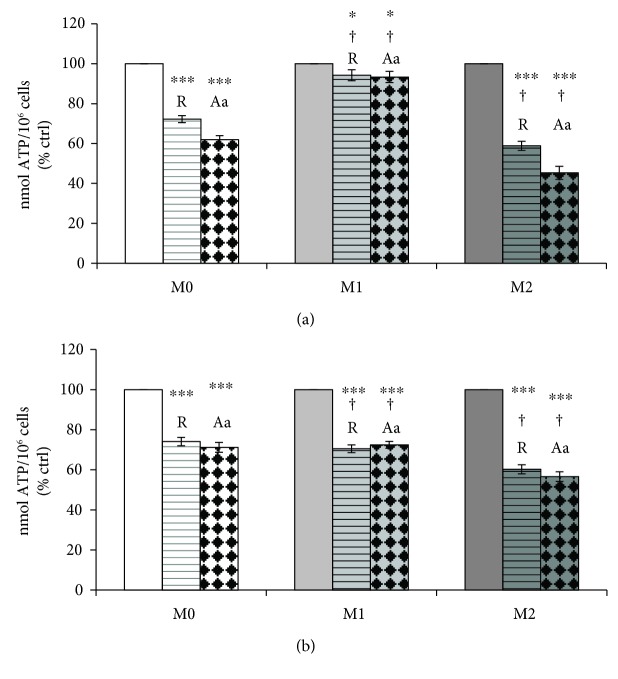
Effect of cocoa polyphenol extract on ATP production by oxidative phosphorylation in macrophage phenotypes. Macrophage-differentiated THP-1 (M0) cells and polarized (M1, M2) cells were incubated in the presence of rotenone (R) and antimycin A (Aa) as indicated under the Materials and Methods. Cellular ATP, expressed as % of control (in the absence of rotenone and antimycin A), was estimated in the absence (a) and in the presence (b) of cocoa polyphenolic extract at concentration 100 *μ*M GAE. Error bars represent data from 3 independent experiments: two-way repeated measures ANOVA (two-factor repetition), followed by Student-Newman-Keuls method. ^∗^*p* < 0.05, ^∗∗∗^*p* < 0.001, cells treated with R and Aa versus untreated. Comparisons M1 versus M2 within treatment: †*p* < 0.001.

**Figure 5:**
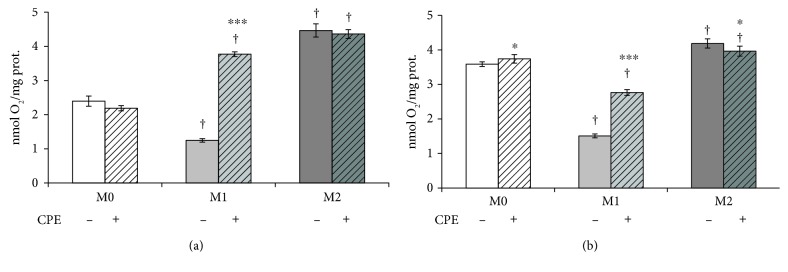
Effect of cocoa polyphenol extract on mitochondrial complex I and complexes II + III in macrophage phenotypes. Macrophage-differentiated THP-1 (M0) cells and polarized (M1, M2) cells were incubated in the absence (−) and in the presence (+) of cocoa polyphenolic extract (CPE) at concentration 100 *μ*M GAE as described under the Materials and Methods. The activities of complex I (a), rotenone sensitive, and complexes II + III (b), antimycin A sensitive, estimated in M0, M1, and M2 phenotypes, through polarografic assay, were expressed as nmol O_2_/mg total proteins. For further details, see under the Materials and Methods. Error bars represent data from 3–5 independent experiments: two-way repeated measures ANOVA (two-factor repetition), followed by Student-Newman-Keuls method. ^∗^*p* < 0.05, ^∗∗∗^*p* < 0.001, cells treated with CPE versus untreated. Comparisons M1 versus M2 within treatment: †*p* < 0.001.

Macrophage metabolism was evaluated through measurement of cellular ATP levels (Figure 4) and of oxygen consumption by mitochondrial complexes (Figure 5) in the differentiated THP-1 (M0) and polarized M1 and M2 cells. The results presented in Figure 4 represent the ATP levels under strict glycolytic condition, in the presence of mitochondrial respiratory chain inhibitors as described under the Materials and Methods.

The results presented in Figure 4(a) show that ATP levels did not decrease in M1 macrophages, in the presence of rotenone and antimycin A. On the other hand, in M0 and M2 cells, they were significantly reduced in the presence of mitochondrial inhibitors. In Figure 4(b), the effect of cocoa polyphenolic extract on ATP levels was estimated in M0, M1, and M2. The results presented in Figure 4(b) indicate that, after cocoa polyphenolic extract treatment, ATP levels decreased in M1 macrophages, in the presence of rotenone and antimycin almost at the same levels of M0 and M1. However, cocoa extract did not influence ATP levels in M0 and M2 macrophages in comparison to untreated cells.

Then, measurement of oxygen consumption was performed for evaluating mitochondrial functionality in macrophage phenotypes. The activities of complex I (Figure 5(a)), rotenone sensitive, and complexes II + III (Figure 5(b)), antimycin A sensitive, expressed as nmol O_2_/mg total proteins, were lower in the M1 macrophages than in M2 cells. The incubation in the presence of cocoa polyphenol extract induced a significant increase of oxygen consumption in M1 by both mitochondrial complexes (Figures 5(a) and 5(b)), thus, suggesting a more oxidative metabolism like phenotype M2. Cocoa treatment had no effect on M0 and M2 macrophages.

## 4. Discussion

This study demonstrates novel findings on the anti-inflammatory role of cocoa and its polyphenols, in vitro in a model of THP-1-derived macrophages. We show, for the first time, that cocoa extract dramatically inhibited the secretion of the proinflammatory cytokines TNF-*α*, IL-6, IL-1*β*, and IL-12 in INF-*γ*/LPS-stimulated macrophages of the same percentage by which it increased cell viability (about 30%), compared to control. More interesting, however, is the finding related to cocoa polyphenol-induced production of the anti-inflammatory cytokine IL-10 in inflammatory M1 macrophages whose levels are similar to those observed in M2 state. Thus, after cocoa treatment, M1 macrophages showed the same levels of IL-12 and IL-10 present in M2 phenotype. In conclusion, following cocoa treatment, M1 macrophages are comparable to M2 cells regarding cytokine production, suggesting that cocoa could promote a shift toward an alternative M2 macrophage phenotype. Our results also demonstrated that cocoa extract influences macrophage metabolism, increasing ATP production through oxidative phosphorylation and O_2_ consumption by mitochondrial respiratory complexes in M1 macrophages. ATP production by oxidative phosphorylation is blocked at the level of NADH dehydrogenase or complex I, which is the first protein in the electron transport chain, by rotenone and at the level of cytochrome c reductase or complex III, which represents the second protonic pump by antimycin A. Both complexes contribute to generate electrochemical gradient across mitochondrial membrane used for the synthesis of ATP by oxidative phosphorylation. In M1 macrophages, in the presence of rotenone and antimycin A, ATP levels do not changed, so M1 phenotype has mainly a glycolytic metabolism as confirmed by others [16]. On the other hand, ATP levels are reduced in M2 cells in the presence of mitochondrial inhibitors in a significant fashion, thus, suggesting that an aliquot of cellular ATP has been produced by oxidative phosphorylation and, therefore, M2 phenotype exhibits a more oxidative metabolism. Once identified M1 and M2 glycolytic/oxidative metabolism, M1 macrophages produced more ATP through oxidative phosphorylation after cocoa polyphenol treatment. This reduction of glycolytic ATP in M1 cells suggests that this phenotype acquires a more oxidative metabolism in the presence of cocoa polyphenols, similar to that of M2 phenotype. However, cocoa extract does not influence ATP levels both in M0 and M2 macrophages.

Measurement of oxygen consumption, performed for evaluating mitochondrial functionality in macrophage phenotypes, in particular, activity of complex I, rotenone sensitive, and complexes II + III, antimycin A sensitive, suggests that M1 macrophages show a lower oxygen consumption by mitochondrial respiratory chain. Therefore, M1 cells show a lower respiratory capacity and high levels of glycolytic ATP. Conversely, the mitochondrial complexes I and II + III consumed more oxygen in M2 macrophages, indicating that these cells have a good respiratory capacity. In the presence of cocoa, oxygen consumption by complex I is similar in M1 and M2 cells. Cocoa treatment has no effect on M0 and M2 macrophages. This suggests that cocoa flavonoids, due to their antioxidant activity, act mainly at the level of complex I, which is redox sensitive. Therefore, cocoa polyphenolic extract not only suppresses inflammation in macrophage inflammatory phenotype but also changes macrophage metabolism, promoting oxidative pathways.

The main phenolic compounds present in cocoa extract belong to the flavonoid group, which are powerful antioxidants acting on the inflammatory pathway and immune system [31, 45]. We demonstrate in this study that cocoa flavonoids, when present together as in the cocoa extract, have a notable anti-inflammatory effect in polarized macrophages, as previously demonstrated for other foods, for example, pomegranate juice [20]. Although the concentration in plasma of cocoa flavonoids and their metabolites is known to be low in humans [46, 47], research based on flavonoid-enriched cocoa-derived products with enhanced bioavailability is ongoing [48–50]. In this context, it has been reported that, in healthy subjects, (−) epicatechin reached maximal concentrations of 5.92 ± 0.60 *μ*mol/l 2 h after the consumption of 0.375 g cocoa/kg body wt. of a flavanol-rich cocoa beverage [51]. Furthermore, it must be taken into account that macrophages do not differentiate in the circulation and that data from animal models reported a tissue and/or dose-dependent accumulation [52, 53]. Therefore, our in vitro results suggest an interesting hypothesis for in vivo study design. The anti-inflammatory actions of cocoa polyphenols in vitro, which are demonstrated in this study, are likely due to bioavailability of flavonoids present in cocoa, but the exact mechanism by which they enter into the cells and the molecular pathways involved is unclear. There is some evidence that certain cocoa flavonoids can directly interact with cell signalling and gene expression factors, which regulate expression of many cytokine genes [54, 55]. Further research is needed to shed light on the interactions between cocoa and cell physiology, contributing thus to the body of knowledge of the effects of food compounds on health.

## 5. Conclusions

The present work demonstrated that cocoa polyphenolic extract is able (i) to reduce inflammatory response in M1 macrophage, favoring secretion of anti-inflammatory cytokines; (ii) to induce a phenotypic switch in polarized macrophages, favoring anti-inflammatory or alternative M2-state; (iii) and to influence macrophage metabolism, favoring oxidative pathways. In this work, we demonstrated the anti-inflammatory and metabolic effects of cocoa and its polyphenols on polarized macrophages, indicating polarizing ability of cocoa toward the M2 phenotype. For using cocoa as dietary supplementation or in the prevention of pathologies, more work is needed to better evaluate the effects of cocoa polyphenolic extract on primary cell lines and/or in vivo and to identify pathways or molecular signals involved in the M1/M2 metabolic switch, induced by the cocoa polyphenols extract.

## Figures and Tables

**Table 1:** Chemical properties of cocoa polyphenol extract. GAE, gallic acid equivalent, TE, Trolox equivalent. Data were expressed as mean of five replicates ± standard deviation. Polyphenol extraction yield was calculated as follows: % (*w/w*) = mass in dry basis/mass of initial weight fed for extraction × 100.

Beans weight (g)	Extraction solvent (*v/v*)	Yield (mg polyphenols/g fresh mass)	Polyphenol content (mg GAE/l)	Antioxidant capacity (mM TE/g fresh mass)
3	Me OH/ H_2_O (70 : 20)	170	8.4 ± 0.5	158 ± 1.6

**Table 2:** Identification by HPLC-PDA-MS of cocoa polyphenolic bioactive molecule extract. UV maximum absorption (UV), retention times (t_R_), and m/z ([M-H]−) values of polyphenols identified in cocoa extract were analyzed.

Peak number	Compound	UV	*t*_R_	m/z [M-H]−
1	Unknown	272	1.7	
2	Caffein	272	2.0	195
3	Unknown	210, 270	2.2	387, 453
4	Chrysophanol-hexoside	210, 274	3.3	415, 253
5	Quinic acid	298, 320	3.6	191
6	Vanillic acid derivative	220, 275	4.7	282
7	Unknown	296	5.1	449
8	Theobromina	272	7.5	181
9	Protocatechuic acid	228, 260, 294	8.0	153
10	Unknown	467	9.2	467
11	Cinnamic acid derivative	213, 301, 320	11.2	294
12	Catechin-3-O-glucoside	278	11.4	451, 289
13	Catechin derivate	278	12.0	497, 451
14	Catechin sulphonic acid	274	12.6	369, 289
15	Unknown	294, 298, 307	13.6	407, 305
16	Procyanidin B dimer	278	14.4	577
17	Procyanidin B dimer	278	15.5	577
18	Procyanidin trimer	278	17	865
19	Procyanidin tetramer	278	17.5	1153
20	Clovamide	320	17.9	358
21	Catechin derivative	278	18.6	720
22	Procyanidin B dimer	278	21.5	577
23	Dideoxyclovamide	320	23.8	326, 282

## Abbreviations

Complex I: NADH dehydrogenase
Complex II: Succinate dehydrogenase
Complex III: Cytochrome c reductase
CPE: Cocoa polyphenolic extract
GAE: Gallic acid equivalent
IFN-*γ*: Interferon-*γ*
IL-1*β*: Interleukin 1*β*
IL-10: Interleukin 10
IL-12: Interleukin 12
IL-6: Interleukin 6
LPS: Lipopolysaccharide
PDA: Photodiode array
PMA: Phorbol 12-myristate 13-acetate
RNS: Reactive nitrogen species
ROS: Reactive oxygen species
TEAC: Trolox equivalent antioxidant capacity
TLRs: Toll-like receptors
TNF-*α*: Tumor necrosis factor-*α.*
